# ETS and Learning: Children’s Exposure Linked to Cognitive Effects

**Published:** 2005-01

**Authors:** David C. Holzman

Previous studies have linked exposure to environmental tobacco smoke (ETS) with lower performance on tests of intelligence, reasoning ability, and language development, as well as higher risk for grade retention, suggesting that such exposure may cause cognitive deficits. Other adverse effects linked with ETS exposure include middle ear infections, colic, sudden infant death syndrome, and exacerbation of asthma. New findings now show that even extremely low-level exposure to ETS may be neurotoxic, according to a team led by Kimberly Yolton of the University of Cincinnati College of Medicine and Cincinnati Children’s Hospital Medical Center **[*EHP* 113:98–103]**. In fact, although a dose–response relationship held for all exposures, the greatest deficits proportionally speaking occurred when overall exposure was low, a phenomenon also noted in lead exposure.

The current study is notable for being the largest of its type, including 4,399 children aged 6–16 years who participated in the Third National Health and Nutrition Examination Survey (NHANES III), conducted from 1988 to 1994. It is also the first to rely solely on a biological marker of exposure—serum cotinine—rather than on data from interviews or questionnaires. “Reports of ETS exposure are complicated by poor recall, an inattention to crucial details such as adjustment for the amount of tobacco exposure, the child’s proximity to the smoker, room ventilation, and other factors that may compromise the validity of exposure measures,” the authors write. Furthermore, people tend to underreport smoking, which is increasingly being seen as a socially undesirable behavior.

While participating in NHANES III, children provided blood samples and took the reading and math subtests of the Wide Range Achievement Test–Revised and the block design and digit span subtests of the Wechsler Intelligence Scale for Children–III (the former Wechsler subtest measures visual construction abilities, and the latter, short-term and working memory). For the current analyses, children were excluded from the sample if they had reported using tobacco products within five days of cognitive testing and blood collection, or if their serum cotinine concentration indicated they probably were active smokers.

Yolton and colleagues measured serum cotinine concentrations in the samples and correlated the data with the children’s test scores. The results showed that children exposed to ETS had mildly to moderately depressed scores on tests of math, reading, and visuospatial skills as compared to children who lacked such exposures, but no deficits in memory. “The range of decrement in scores is very roughly equivalent to the loss of two to five IQ points at varying levels of exposure,” says Yolton. The authors estimate that more than 21.9 million U.S. children are at risk for ETS-related reading deficits.

The study is limited by NHANES III’s lack of measures of parental cognitive abilities and quality of home environment. Also, it is unclear whether the serum cotinine levels, taken just once for each subject, represented chronic or acute levels. However, other studies have shown serum cotinine concentrations to be stable in both smokers and nonsmokers. And although more research is needed to confirm these findings, the authors say this analysis adds to the evidence supporting policy to further reduce childhood exposure to ETS.

